# Bayesian Estimation of the True Prevalence of Caprine Arthritis Encephalitis in Hungarian Goat Herds

**DOI:** 10.3390/v17111455

**Published:** 2025-10-31

**Authors:** Krisztina Bárdos, Marietta Máté, Katalin Veres, Zsolt Lang, Giuseppe Bertoni, Carlos Eduardo Abril, Snorre Stuen, Saulius Petkevičius, Marcin Mickiewicz, Michał Czopowicz, Jarosław Kaba, László Ózsvári

**Affiliations:** 1 Department of Veterinary Forensics and Economics, Institute of Economics and Biostatistics, University of Veterinary Medicine Budapest, István u. 2, 1078 Budapest, Hungary; bardos.krisztina@univet.hu (K.B.); mate.marietta@univet.hu (M.M.); 2 National Laboratory of Infectious Animal Diseases, Antimicrobial Resistance, Veterinary Public Health and Food Chain Safety, University of Veterinary Medicine Budapest, István u. 2, 1078 Budapest, Hungary; 3 Department of Biostatistics, Institute of Economics and Biostatistics, University of Veterinary Medicine Budapest, István u. 2, 1078 Budapest, Hungary; veres.katalin@student.univet.hu (K.V.); lang.zsolt@univet.hu (Z.L.); 4 Institute of Virology and Immunology, Department of Infectious Diseases and Pathobiology, Vetsuisse Faculty, University of Bern, Laenggass-Str. 122, 3012 Bern, Switzerland; giuseppe.bertoni@unibe.ch (G.B.); carlos.abril-gaona@unibe.ch (C.E.A.); 5 Department of Production Animal Clinical Sciences, Norwegian University of Life Sciences, Svebastadveien 112, 4325 Sandnes, Norway; snorre.stuen@nmbu.no; 6 Department of Veterinary Pathobiology, Veterinary Academy, Lithuanian University of Health Sciences, Tilzes Str. 18, 47181 Kaunas, Lithuania; saulius.petkevicius@lsmu.lt; 7 Division of Veterinary Epidemiology and Economics, Institute of Veterinary Medicine, Warsaw University of Life Sciences-SGGW, Nowoursynowska 159c, 02-776 Warsaw, Poland; marcin_mickiewicz@sggw.edu.pl (M.M.); michal_czopowicz@sggw.edu.pl (M.C.); jaroslaw_kaba@sggw.edu.pl (J.K.)

**Keywords:** Bayesian model, small ruminant, goat, lentivirus, caprine arthritis-encephalitis, herd-level seroprevalence, within-herd seroprevalence, true prevalence

## Abstract

**Background:** Caprine arthritis encephalitis (CAE) is a major viral disease of goats, caused by small ruminant lentivirus (SRLV), associated with chronic arthritis, mastitis, pneumonia, and encephalitis, leading to economic losses and reduced animal welfare. This study aimed to estimate the true prevalence of CAE in Hungarian goat herds, based on nationwide sampling and statistical modeling. **Methods:** Blood samples from 1218 goats in 53 herds were tested using ELISA, and true prevalence was estimated by Bayesian analysis. **Results:** The mean herd true prevalence (HTP) was 29.1% (95% CrI: 20.8–38.5%), while within the infected herds, the conditional within herd prevalence (CWHP) reached 58% ± 27.1%. Medium- and large-sized herds (>50 animals) showed the highest mean HTP (77.8% and 74.9%, respectively). No significant regional differences were observed, indicating that CAE is uniformly distributed across the country. **Conclusions:** Our findings place Hungary among moderately to highly affected European countries and highlight the need for a nationwide control strategy integrating routine serological surveillance, biosecurity improvements, farmer education, and long-term tools such as selective breeding.

## 1. Introduction

Small ruminant lentivirus (SRLV) genotypes A and B correspond to *maedi-visna virus* (MVV) and *caprine arthritis-encephalitis virus* (CAEV), respectively. These viruses cause chronic, multisystemic inflammatory diseases that significantly impair sheep and goat production and represent a major source of economic loss [1,2]. The CAEV from the *Lentivirus* genus was first described in the USA in the mid-1970s and successfully isolated in 1980 [3,4]. The virus is the causative agent of a chronic disease known as caprine arthritis encephalitis (CAE), which causes mastitis in adult goats [5,6,7], chronic progressive arthritis [8], and leuko-encephalomyelitis in young goats [9]. Thus, the disease causes significant economic losses in the goat industry worldwide, resulting in decreased milk production, shorter lactations, increased somatic cell counts, reduced growth rates and longevity, reproductive disorders, premature culling, and increased incidence of other diseases [5,6,10,11,12,13].

Direct transmission of SRLV infection can occur through contact, body fluids, faeces, virus-contaminated colostrum or goat milk [14,15]. As symptoms develop slowly and only in a proportion of infected goats [8,16], SRLVs infection spreads in the herd long before goats showing the first symptoms are noticed. Therefore, serological screening of the herd is the only method for early detection of the disease [17]. Vaccines or treatment against CAE are ineffective and supportive therapy is often costly [18,19]. Diagnostic strategies mainly rely on enzyme-linked immunosorbent assay (ELISA) for herd-level screening, supported by molecular methods such as polymerase chain reaction (PCR), which provide high sensitivity (Se) and specificity (Sp) for rapid detection [20,21]. However, the marked genetic variability and subtype diversity of SRLVs undermine diagnostic accuracy and complicate eradication efforts [22]. Reviews consistently emphasize that program success depends on reliable test performance and sustained test-and-remove schemes, which remain challenging in many regions [12,23].

International studies consistently identify herd size, age, and management practices as the major risk factors for SRLV infection. Larger herds are repeatedly associated with higher prevalence rates, reflecting the role of increased stocking density and frequent animal movement in viral transmission [24,25,26,27]. Age-related accumulation of infection has also been observed, with Iraqi goats over five years old reaching 40% prevalence and older sheep also showing significantly higher infection rates [28]. Similar age-related trends were seen in Turkey, where sheep had higher prevalence than goats, and older animals (>48 months) were at elevated risk [29]. Several studies highlighted the influence of management practices and biosecurity. Portuguese herds with intensive production systems, dairy specialization, large herd sizes (>100 animals), and frequent participation in livestock competitions showed markedly higher infection levels [30]. In Brazil, the importation of bucks, lack of isolation for sick animals, and participation in fairs were identified as major risk factors [31], while another Brazilian study confirmed that imported bucks carried higher infection rates [32].

SRLVs are documented across all continents where goats and sheep are raised. A global meta-analysis reported individual-level prevalences averaging 40.9% in Europe, 16.7–21.8% in Asia and North America, and as low as 1.7% in South and Central America [33]. Today, in many countries, various surveillance and monitoring systems are playing an increasingly important role to ensure a high level of animal health and welfare, and product safety [34]. In Switzerland, because of the mandatory national control program, which started in 1984, CAE seroprevalence was reduced to 0.38% at the herd level and to 0.06% of animal level in about 25 years (Census 2011–2012), and clinical disease was eliminated [35]. The Swiss goat population is now considered free of CAEV (SRLV-B), but MVV (SRLV-A) is still circulating at a low level. This represents a considerable improvement since 1984, when CAEV seroprevalence in the Swiss goat population was around 60–80% [11,35,36]. In the Netherlands, a similar system has been in place since 2003, whereby annual data analysis of the health of small ruminants in the Netherlands is carried out to retrospectively monitor trends and developments in goat health and welfare [34]. In contrast, Norway implemented a comprehensive eradication program (“Healthier Goats Project”) targeting lentivirus diseases in goat herds from 2001 to 2014, resulting in effective elimination of CAE in participating herds [37]. This program also had a positive economic impact on dairy goat farmers within a decade [38,39]. In South Tyrol, Italy, a compulsory CAEV eradication campaign was initiated in 2007. This program effectively eliminated clinical disease and substantially reduced seroprevalence at both herd and individual levels, though the complete eradication has been hindered by a persistent ‘tailing phenomenon’ with re-emerging infections in sanitized herds [40,41].

In contrast to some countries where comprehensive SRLV surveillance programs are lacking, Hungary has limited data available regarding small ruminant lentivirus prevalence. A molecular study conducted in 2023 investigated SRLV genotypes in ten previously seropositive goat herds, revealing infection in all herds and detecting both genotypes A and B, with 81.5% of seropositive goats testing PCR-positive [42]. Earlier serological surveys in Hungary also indicated that approximately 30% of goats were seropositive for CAEV [43]. In addition, a selection-based eradication program implemented between 2020 and 2023 in a 150-doe dairy goat herd successfully reduced the proportion of seropositive animals from up to 100% to below 20%, though complete eradication was hindered by delayed seroconversion and re-emergence of infections [44].

The aim of this study was to estimate the true prevalence of CAE in the Hungarian goat herds, namely: (1) the proportion of truly infected herds within all herds (HTP—herd level true prevalence) and (2) the proportion of infected animals within infected herds (CWHP—conditional within herd animal-level prevalence).

## 2. Materials and Methods

### 2.1. Sampling and Serological Testing

The study was conducted between June 2022 and February 2023 and included a survey of 53 dairy and meat goat herds across all seven NUTS regions of Hungary. The participation was voluntary, and informed consent was obtained from each herd owner. The inclusion criteria for goat herds participating in the study were providing access for blood sampling and data collection, and ensuring safe handling of animals during sampling. For each herd, a sample submission form was used to record all relevant data and ensure accurate sample tracking. The header section of the form captured administrative details, including the herd location, date of sampling, the farm owner/administrator, the name of the attending veterinarian and the individual responsible for sample collection. Each herd was assigned a unique project identification code for traceability. The main section of the form contained a structured table where data for each sampled animal were recorded in separate rows. Information collected included the sample identifier, the animal’s official identification number, sex (male or female), and clinical observations indicating the presence or absence of joint swelling and superficial abscesses, which were marked as “yes” or “no”.

Blood samples were collected from identifiable adult male and female goats (>1 year old) of any breed, with the number of samples collected from each herd proportional to the herd size. Blood was collected from the jugular vein into 10 mL BD Vacutainer^®^ Serum Tubes with clot activator (Ref. 367896; Becton, Dickinson and Company, Franklin Lakes, NJ, USA) using 18 G (1.2 × 40 mm) needles (B. Braun Melsungen AG, Melsungen, Germany), without shaving or disinfecting the skin. According to Hungarian legal regulations [45], no specific ethics approval was required for blood collection from farm animals.

A total of 1218 samples were collected and tested for antibodies against MVV/CAEV using the ID Screen^®^ MVV/CAEV Indirect ELISA (ID.vet Diagnostics, Grabels, France) in the laboratory of the University of Veterinary Medicine Budapest (UVMB), following the manufacturer’s instructions. After clotting, serum was separated, transferred into Eppendorf tubes, and stored at −20 °C until ELISA analysis.

For the ELISA procedure, samples and controls were added to the microplate wells. In the presence of anti-MVV/CAEV antibodies, antigen–antibody complexes formed, which subsequently bound to an anti-ruminant horseradish peroxidase (HRP) conjugate, creating antigen–antibody–HRP complexes. After washing to remove unbound conjugate, the substrate solution (TMB; Sarstedt AG & Co. KG, Nümbrecht, Germany) was added. Color development depended on the antibody content of the sample: a blue color (turning yellow after addition of the Stop Solution) indicated antibody presence, whereas no color indicated absence. Optical density (OD) was measured at 450 nm.

The test was validated if the mean OD of the positive control (ODPC) exceeded 0.350 and the ODPC/ODNC (negative control) ratio was greater than 3.

For each sample, the sample-to-positive percentage (S/P%) was calculated:

- S/P ≤ 50%: negative,
- 50% < S/P < 60%: inconclusive,
- S/P ≥ 60%: positive.

For prevalence estimation, samples with S/P% between 50% and 60% were classified as inconclusive. At a threshold of 50% S/P, the test demonstrated a Se of 91.7% (95% CI: 85.0–95.6%) and Sp of 98.9% (95% CI: 96.2–99.7%) [46,47].

### 2.2. Statistical Model

#### 2.2.1. Description of Model Components

The proportion of positive cases (apparent prevalence, AP) differs from the true prevalence due to false positive and false negative test results. We aimed to estimate both the proportion of truly infected herds within all herds (herd-level true prevalence, HTP), and the proportion of infected animals within infected herds (conditional within herd animal-level prevalence, CWHP), taking into account that the diagnostic test is not perfect. A Bayesian hierarchical model, including herds as random clusters, was fitted to the animal-level serum ELISA test results obtained from goats randomly and independently sampled within each herd.

We assumed that the true prevalence of infection across herds is distributed around the regional mean prevalence (µ), and that the true prevalence of infection within each herd (${CWHP}_{i})\;$ also depends on unobserved herd-specific factors. Random herd effects are modeled as normally distributed variables and quantified by their variance. The proportion of truly infected herds among all herds (HTP) is related to the true within herd prevalences (${CWHP}_{i})\;$ through the apparent prevalence (AP_i_) and the sensitivity and specificity of the diagnostic test. A more detailed description of the model is provided below.

A positive test for a goat from a herd can be attributed to (i) either the herd being infected with a probability of HTP and the test giving a positive result with a probability of the apparent prevalence (AP) in that herd, or (ii) the herd not being infected with a probability of (1-HTP) and the test incorrectly identifying the goat as positive with a probability of (1-Sp).

Let N_i_ be the number of goats selected in the sample in the i-th herd. Let Pos_i_ be the number of test positive animals among the N_i_ animals sampled. The probability distribution of Pos_i_ is modelled to be the mixture of two binomial distributions:

(1) $${Pos}_{i}\sim HTP\times Binomial\left({N_{i},AP}_{i}\right)+\left(1-HTP\right)\times Binomial\left(N_{i},1-Sp\right).$$

Here, HTP is the herd-level true prevalence and AP_i_ is the conditional within-herd apparent prevalence; that is the probability that a randomly selected goat in herd i tests positive, given herd i is infected.

The first part of the sum above applies when the i-th herd is assumed to be infected and the second part describes the case when the herd is free of infections. AP_i_ is further modelled as the sum of the probabilities of true positive and false positive test result:

(2) $${AP}_{i}=Se\times {CWHP}_{i}+\left(1-Sp\right)\times (1-{CWHP}_{i})$$

with ${CWHP}_{i}$ the conditional within herd animal-level prevalence in herd i and Se, Sp the sensitivity and specificity of the serum ELISA test.

The spread of ${CWHP}_{i}$ around its mean µ, representing heterogeneity of prevalence of infection between infected herds is modelled by a beta distribution with mean μ, and variance $\mu \left(1-\mu \right)/\left(1+\psi \right)$, where ψ is the precision parameter [48].

This modeling framework was further refined by allowing for different HTP and μ parameters depending on herd size (very small ≤ 20, small 21–50, medium 51–100, large ≥ 101 goats). Moreover, to assess the magnitude of regional heterogeneity, a modified model with regional HTP and µ parameters following beta distributions was investigated. Pairwise comparisons were carried out between the effects of herd size categories. For regions, the difference between the overall mean and the individual regional effects was assessed.

#### 2.2.2. The Choice of Priors

Bayesian methodology integrates the observed data with a prior distribution that reflects prior experience and expert knowledge. The prior distribution is refined and updated using the actual data to obtain the Bayesian posterior estimates of the parameters of interest [49].

Non informative (vague) priors were applied to HTP, μ, and ψ. More specifically,

(3) HTP~Beta(1,1),

(4) μ~Beta(1,1),

(5) ψ~Gamma(0.01,0.01),

where Beta and Gamma are the beta and gamma distributions, respectively.

The Se and Sp were estimated in [46]. Based on this estimate, ref. [24] used the informative priors

(6) Se~Beta(97.0,9.2),

(7) Sp~Beta(86.0,1.27)

which we adopted literally in our analysis.

The Bayesian model was implemented using the rstan package of R 4.1.3 statistical software [50,51]. The R and STAN source codes of the Bayesian model, including an illustrative data simulating code are available as Supplementary Material.

#### 2.2.3. Model Convergence Diagnostics

We ran four chains of 10,000 Markov Chain Monte Carlo (MCMC) iterations each, starting from different sets of initial values of the parameters of the model. The first 5000 warm-up iterations were discarded in each chain. Convergence was checked and demonstrated by visual inspection of the trace plots of the chains and was quantified using the split R-hat formula [49].

#### 2.2.4. Posterior Predictive Check of Model Fit

To evaluate the fit of our Bayesian model to the observed data, replicates of counts of positive tests were simulated for each herd from the posterior distributions of the model and evaluated versus the observed counts. The discrepancy of the distributions of replicated and observed counts was checked by visual inspection using smoothed histograms. Bayesian *p*-values comparing means, medians, and standard deviations of observed and replicated counts were computed and evaluated. All the *p*-values fell within the range of 0.39–0.63, demonstrating good fit of the model [49]. All the above was performed using the R package bayesplot version 1.14.0 [52].

## 3. Results

### 3.1. Descriptive Statistics

**Figure 1:**
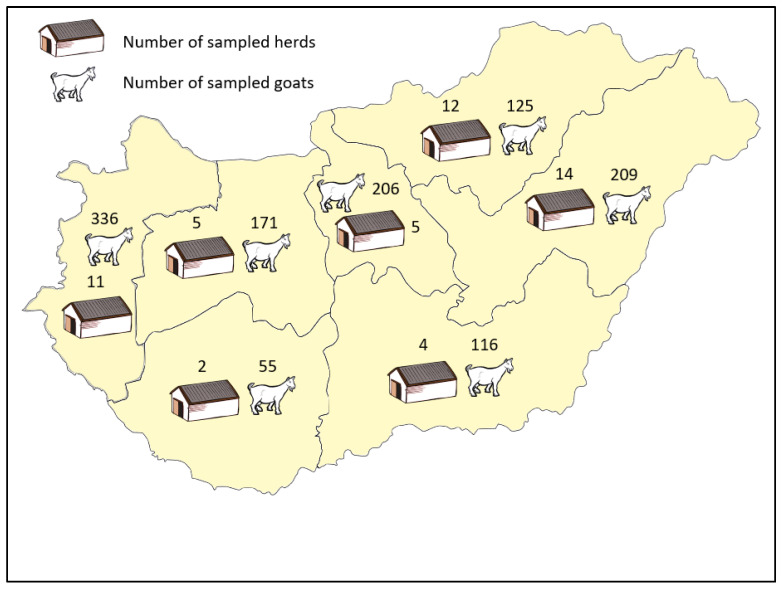
Geographic distribution of herds, number of goats sampled in Hungary.

We collected and tested blood samples from goats older than 12 months. There were 1218 sampled animals among 2404 eligible goats from 53 herds in Hungary. The median herd size and the median sample size per herd of eligible population was 19 (interquartile range: 8–65) and 19 (interquartile range: 8–30), respectively. The geographic distribution of herds with the corresponding sample size is shown in Figure 1.

**Figure 2:**
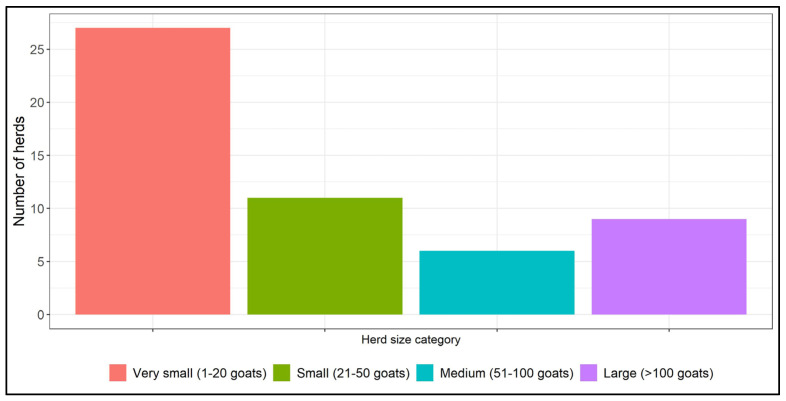
Distribution of herd size by number of goats older than 12 months.

The number of herds in the herd size categories is shown on Figure 2.

In total, 373 goats tested positive, resulting in an overall within-herd apparent prevalence of 30.6%. The number of goats and the ELISA status by herd size categories is shown in Table 1.

There were 25 herds without test-positive goats, which corresponds to a herd-level apparent prevalence of 52.8% (28/53). The number of farms with an apparent animal-level prevalence of >5%, >25%, >50%, and >75% was 20 (37.7%), 12 (22.6%), 9 (17.0%), and 4 (7.6%), respectively.

### 3.2. Bayesian Model

#### Investigation of the Effects of Herd Size and Regions

The estimate for mean HTP within the herd size categories and for mean CWHP is shown in Table 2. The average of HTPs weighted by the number of sampled herds belonging to each category is 29.1% (95% credible intervals [CrI]: 20.8–38.5%).

**Figure 3:**
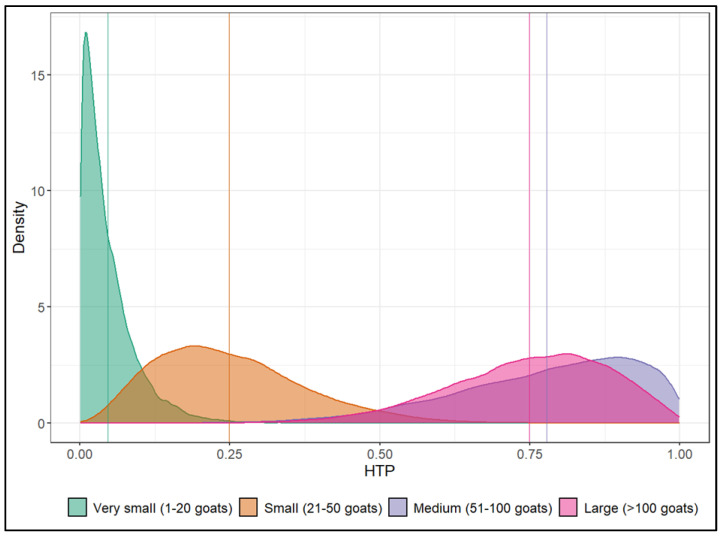
Posterior probability density functions and mean values for herd-level true prevalence (HTP) by herd size category.

We evaluated the 95% CrI for pairwise differences in HTPs related to each pair of herd size groups. We found that only the CrIs for very small and small herds, and for medium and large herds contained zero. Consequently, regarding HTP, very small and small herds (≤50 goats) and medium and large herds (≥51 goats) may be merged to form two homogeneous groups. The posterior distribution of HTPs by herd size category is shown on Figure 3.

**Figure 4:**
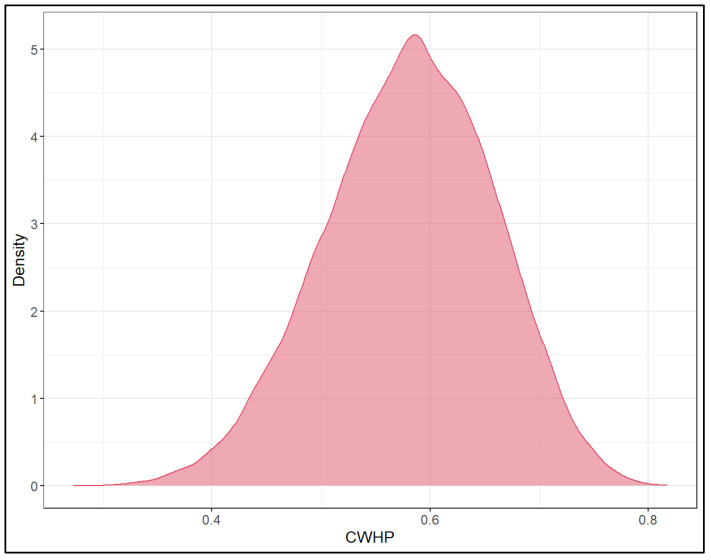
Posterior probability density functions for conditional within herd animal-level prevalence (CWHP).

We modelled conditional within herd prevalence (CWHP) applying beta distribution (Figure 4). The posterior mean µ and precision ψ of this distribution was estimated 0.580 (0.423–0.722), and 2.307 (1.110–4.555), respectively. Consequently, the CWHP was 58.0% ± 27.1% (mean ± SD).

**Figure 5:**
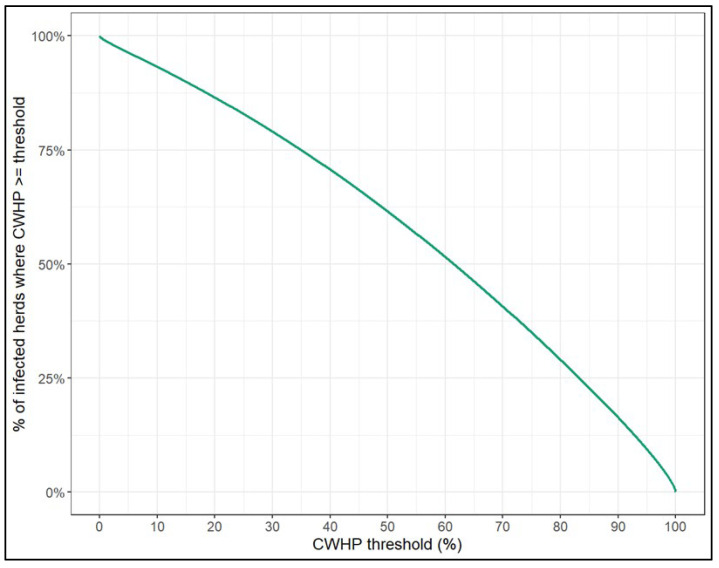
Posterior probability of conditional within herd animal-level prevalence (CWHP) being at least a given threshold.

The proportion (i.e., posterior probability) of infected herds having at least 25%, 50% or 75% true prevalence was 82.9%, 61.6% and 35.0%, respectively (Figure 5).

Additionally, we estimated and compared mean CWHP in each size category. All the 95% CrIs of their pairwise differences contained zero, therefore we reduced the Bayesian model to fit a single beta distribution to CWHP of all herds.

For regions, the difference between the overall mean and the individual regional HTP and CWHP was assessed. We found that all the 95% CrIs of the effect differences contained zero, i.e., no substantial effect of the regions was detected on HTP and CWHP.

## 4. Discussion

### 4.1. Prevalence and Within-Herd Dynamics of CAE

This study represents a large-scale, nationwide survey of CAE prevalence in Hungary, providing an overview of the current epidemiological status of the infection in the national goat population. The results confirm that CAE is widespread across Hungarian goat herds and represent a challenge for herd health management [53].

In this study, 52.8% of surveyed herds (28/53) contained seropositive animals (AP). This places Hungary in the mid-to-high prevalence range internationally, comparable to Lithuania (57%) [54] and Poland (26.3%) [55], but lower than several countries such as Portugal (86%) [30], Albania (66.7%) [56], and Taiwan (98.5%) [57]. However, the studies from Portugal and Albania were conducted in selected regions rather than nationwide, which may have influenced the reported prevalence [30,56]. CAEV prevalence in parts of Asia and the Middle East is generally lower, with Japan (15%) [25], Thailand (31%) [26] and Jordan (23.2%) [15], reporting reduced infection levels. Switzerland achieved near-zero prevalence (0.38%) through a nationwide eradication program [35]. Brazilian surveys show highly variable results, with reported prevalence ranging from 35% [32] to 67.1% [31]. Studies in Brazil and Japan used the Agar Gel Immunodiffusion Assay (AGID) instead of ELISA, which may have underestimated infection levels. While ELISA is more sensitive than AGID and is widely used for routine screening, it can still misclassify infected animals, particularly in early infection stages before SRLV-specific antibodies are detectable or in cases of delayed seroconversion [58].

Our Bayesian model estimated the herd-level true prevalence of CAE in Hungary at 29.1% (95% CrI: 20.8–38.5%), indicating a substantial national burden of infection and placing Hungary in the mid-range of prevalence reported in other European countries, thereby reinforcing the value of Bayesian modeling for providing accurate prevalence estimates. Comparable estimates have been reported in Poland, where a large-scale study using Bayesian modeling calculated a 33.3% HTP (95% CI: 26.5–38.2%) of SRLV in sheep herds [59], supporting the robustness of this approach for prevalence estimation. Another large-scale Polish survey focusing on CAE in goats reported an even higher HTP of 61% (95% CI: 53–68%), highlighting considerable variation between species and production systems [24]. Similarly, Lithuania reported a 56% HTP of CAE (95% CrI: 36–76%) [54], while a survey from Thailand estimated a 31% true herd-level prevalence of CAE based on competitive ELISA testing [26], aligning closely with our findings.

The overall within-herd apparent prevalence of CAE in Hungary was 30.6%, placing the country in a mid-to-high range of infection. This value was lower than the herd-level apparent prevalence (47.2%), which also observed in other countries, such as Japan [25], Thailand [26], Portugal [30], Brazil [31,32], Jordan [15], Taiwan [57], Philippines [60] and Switzerland [35]. Switzerland stands out as a success story, achieving near eradication with 0.06% animal-level prevalence after decades of strict CAE control measures [11,35]. Compared with other countries, Hungary’s within-herd prevalence is similar to levels reported in Poland (33.3%) [55], Spain [6] and Croatia [61], likely reflecting comparable herd structures and diagnostic approaches (ELISA). Higher prevalence values reported in Portugal [30] and Mexico (78% PCR-positivity) [62] may be linked to intensive dairy production systems, frequent animal movement, and larger herd sizes. In contrast, lower prevalence in Iraq [28], Turkey [29], Somalia [63], Korea [64] and Malaysia (in deer) [65] may result from smaller, less intensive herds, regional underreporting, or differences in diagnostic Se.

The conditional within-herd prevalence in infected Hungarian herds was estimated at 58.0% ± 27.1%, indicating that once SRLV is introduced into a herd, it spreads rapidly, affecting the majority of animals. This is consistent with findings from Poland, where the median CWHP reached 42% (IQR: 17–84%) [24], further confirming the high infection pressure observed in infected goat populations. In contrast, studies on other species, such as SRLV seroprevalence in deer in Malaysia, reported much lower infection levels (8.4%) [65].

### 4.2. Risk Factors of CAE Infection

Our analysis revealed a strong association between herd size and CAE prevalence in Hungary, with medium- and large-sized herds (>50 animals) showing the highest HTP (77.8% and 74.9%, respectively). This pattern is consistent with multiple international studies, underscoring herd size as a key determinant of infection risk. In Poland, herd size has been repeatedly identified as a major determinant of infection risk, with medium and large herds reporting true herd-level prevalence between 72% and 86% [24,27]. Longitudinal data from the same region suggest that SRLV infection accumulates over time, with older goats reaching prevalence levels of 55.7% [55], and that herd size remains a reliable predictor of infection even where regional prevalence varies [59].

Similar associations have been reported elsewhere. In Jordan, larger herds were twice as likely to be infected, and herd-to-herd contact or the addition of new animals further increased risk [15]. In Somalia, age was the strongest predictor, but larger herds and mixed-species farming also contributed to higher prevalence [63]. Nyi Li et al. [26] confirmed that herd size, herd type, goat movement, and age over three years were key risk factors in Thailand. Research from Japan also showed that farms with more than 10 goats, especially dairy or breeding herds, had significantly higher infection rates [25]. An Albanian study highlighted that production systems and farm-level biosecurity strongly influence transmission of SRLV, and that goats are more susceptible than sheep [56]. While herd size and age consistently emerge as primary risk factors of CAE infection, other variables, such as herd grouping, appear less influential [65].

### 4.3. Control of CAE

Our study demonstrates that CAE is widespread in Hungary, with a high infection pressure at both the herd and individual animal levels. Furthermore, no significant regional differences were detected in herd-level or within-herd prevalence, suggesting that CAE is uniformly distributed across goat-keeping regions. The relatively small herd sizes in our study (median: 19 goats) did not prevent widespread infection, indicating that supposedly frequent animal movements, the absence of structured testing programs, and the lack of coordinated control efforts may be key drivers of virus persistence and spread in Hungary. Therefore, our findings on CAE prevalence underscore the need for a nationally coordinated control program rather than local or farm-level interventions.

Experiences from other countries demonstrate that eradication is achievable but requires sustained effort and significant resources. Switzerland’s national program combined multi-step diagnostic testing (ELISA, Western Blot, SU5 ELISA) with the mandatory culling of SRLV-B-positive goats and their offspring [11,35]. Later adjustments introduced a risk-based surveillance approach, which maintained low prevalence and revealed that SRLV-A strains are less virulent and inefficiently transmitted, allowing for a more targeted approach [36]. In contrast, Hungary’s prevalence places it among moderately to highly affected countries, with considerably higher infection pressure than in regions with long-term control programs. The eradication program in South Tyrol, Italy, showed that strain-specific diagnostics and multi-ELISA testing were essential for sustained control [40]. Norwegian and Portuguese initiatives further highlight that management practices (e.g., shared pastures) and economic feasibility shape program outcomes, while farmer education and engagement are central to success [30,66].

The challenges of farm-level eradication were illustrated by a four-year Hungarian case (2020–2023), in which quarterly ELISA testing, confirmatory PCR, segregation, and artificial kid-rearing initially reduced prevalence but failed to eliminate infection, which subsequently rebounded to 60–87% following outbreaks and stress-related immunosuppression [44]. Similar difficulties have been reported in Thailand, where a multi-pronged approach combining test-and-cull strategies, strict hygiene, and biosecurity was evaluated alongside ELISA, PCR, and virus isolation [11,12,67].

Our results show that CAE is uniformly distributed across Hungarian regions, reflecting the absence of systematic surveillance. Comparable findings from Brazil, Lithuania, and Poland emphasize the role of uncontrolled animal movement and sourcing animals from multiple farms, leading to high herd-level prevalence and co-circulation of SRLV genotypes [31,54,55]. Farmer awareness is consistently low; a Polish study found that arthritis often went unnoticed even in heavily infected populations (median CWHP: 34.6%), which highlights the risk of silent spread and the need for proactive, structured testing [68].

Successful CAE control and eradication strategies must integrate multiple tools. Biosecurity remains central, with key measures including the purchase of only seronegative animals, frequent testing (ELISA, PCR), separation of infected and uninfected goats, removal of infected animals, avoidance of pooled milk feeding, heat treatment of colostrum, and strict disinfection practices [17,31,54,56]. Diagnostic advances are essential due to viral genetic variability, and studies from Greece and Morocco recommend using PCR and next-generation sequencing (NGS) alongside serology to improve strain detection and characterization [69,70]. Selective breeding for CAE resistance is a promising long-term approach, although heritability is low [18].

In summary, our findings reinforce the need for a structured, nationwide CAE control program in Hungary. Such a program should combine routine serological screening with molecular confirmation [71,72], enhanced biosecurity, targeted culling, farmer education, and long-term strategies such as selective breeding [41].

### 4.4. Limitations

Although our large-scale, nationwide survey included herds from all major goat-keeping regions of Hungary, the sample may not fully represent the entire national goat population; therefore, potential regional differences may have been underestimated. The use of ELISA, while widely accepted for routine screening, has inherent diagnostic limitations, including the risk of false negatives during early infection or delayed seroconversion. As a cross-sectional study, our results provide prevalence estimates but do not capture the temporal dynamics of infection or transmission. The Bayesian approach allowed for robust estimation of herd-level true prevalence, yet the relatively small number of herds in some size categories resulted in wide CrI. Future studies employing larger sample sizes, longitudinal designs, and combined serological and molecular methods could yield a more comprehensive understanding of the epidemiology and strain diversity of SRLV in Hungary.

## 5. Conclusions

Our nationwide survey confirms that CAE is widespread in Hungary, with uniformly high prevalence across goat-keeping regions. Herd size was identified as the main risk factor, consistent with findings from other countries. The lack of structured testing, surveillance, and coordinated control likely explains the persistence of infection despite relatively small herd sizes. International experiences show that eradication is possible but resource-intensive, and farm-level efforts are difficult to sustain without national coordination. Therefore, a Hungarian CAE control program should integrate routine serological surveillance with molecular confirmation, biosecurity improvements, farmer education, and long-term tools such as selective breeding. These measures, adapted to the Hungarian production system, are essential for reducing infection pressure and to improve both animal health and farm productivity.

## Figures and Tables

**Table 1:** Apparent prevalence by herd size category in the sampled goat population (n = 1218).

	Very Small (1–20 Goats)	Small (21–50 Goats)	Medium (51–100 Goats)	Large (>100 Goats)	Overall
**Number of tests**	231	273	181	533	1218
Negative	225	227	115	278	845
Positive	6	46	66	255	373
**Apparent prevalence (%)**	2.6%	16.9%	36.5%	47.8%	30.6%

**Table 2:** Posterior mean and 95% credible intervals (CrI) of herd-level true prevalence (HTP) by herd size categories, and conditional within herd animal-level prevalence (CWHP) (%).

Herd Size	Mean	2.5%	97.5%
**HTP—Very small herds** **(≤20)**	4.7	0.2	16.5
**HTP—Small herds** **(21–50)**	24.9	6.0	52.8
**HTP—Medium herds** **(51–100)**	77.8	43.7	98.6
**HTP—Large herds** **(>100)**	74.9	45.8	96.1
**CWHP**	58.0	42.3	72.2

## Data Availability

The raw data supporting the conclusions of this article will be made available by the authors on request.

## Supplementary Materials

The following supporting information can be downloaded at: https://www.mdpi.com/article/10.3390/v17111455/s1, Title: CAE_basic_model.stan, CAE_simulated_data.R.

## Abbreviations

The following abbreviations are used in this manuscript:

SRLV	Small ruminant lentivirus
CAEV	Caprine arthritis-encephalitis virus
MVV	Maedi-visna virus
CAE	Caprine arthritis encephalitis
ELISA	Enzyme-Linked Immunosorbent Assay
PCR	Polymerase chain reaction
UVMB	University of Veterinary Medicine Budapest
HRP	Horseradish peroxidase
TMB	Substrate solution containing 3,3′,5,5′-Tetramethylbenzidine
OD	Optical density
ODPC	Optical density of the positive control
ODNC	Optical density of the negative control
S/P%	Sample-to-positive percentage
HTP	Herd-level true prevalence
CWHP	Conditional within-herd prevalence
AP	Apparent prevalence
Sp	Specificity
Se	Sensitivity
MCMC	Markov Chain Monte Carlo
CrI	Credible intervals
AGID	Agar Gel Immunodiffusion Assay
NGS	Next-generation sequencing
